# Computer-assisted horizontal translational osseous genioplasty: a simple method to correct chin deviation

**DOI:** 10.1186/s40902-020-00278-z

**Published:** 2020-10-20

**Authors:** Seied Omid Keyhan, Abbas Azari, Parisa Yousefi, Behzad Cheshmi, Hamid Reza Fallahi, Mohammad Amin Valipour

**Affiliations:** 1 CMFRC, National Advance Center for Craniomaxillofacial Reconstruction, Tehran, Iran; 2 Craniomaxillofacial Research Center, Tehran University of Medical Sciences, Tehran, Iran; 3 Maxillofacial Surgery and Implantology Research Foundation, Tehran, Iran; 4 Department of Removable Prosthodontics, Faculty of Dentistry, Medical Sciences/University of Tehran, Tehran, Iran; 5 College of Dentistry, Isfahan University of Medical Sciences, Isfahan, Iran; 6 Faculty of Dentistry, Boroujerd Islamic Azad University, Boroujerd, Iran; 7 School of Advanced Technologies in Medicine, Shahid Beheshti University of Medical Sciences, Tehran, Iran; 8 Dental Research Center, Research Institute of Dental Sciences, Shahid Beheshti University of Medical Sciences, Tehran, Iran; 9 Faculty of Dentistry, Ahvaz Jundishapur University of Medical Sciences, Ahvaz, Iran

**Keywords:** Genioplasty, Chin deviation, Translational osseous genioplasty, Computer-assisted, Mandible

## Abstract

**Background:**

Different genioplasty techniques are applied for the adjustment of chin area deformities such as chin deviation.

**Results:**

Thirty patients with simple facial asymmetry due to chin deviation underwent computer-assisted horizontal translational osseous genioplasty. In this technique, a surgical guide was used to cut a bone strip from the side where the chin should be transferred to; then, the same bone strip was used for the filling of the gap that was formed on the opposite side.

**Conclusion:**

According to the experience gained from this study, the authors believe that computer-assisted horizontal translational osseous genioplasty is a simple and reliable technique for patients with facial asymmetry due to chin deviation.

## Introduction

Facial asymmetry can be defined as the lack of correspondence in size, shape, and relative position of parts on two sides of the facial median plane [1]. Different components of facial hard and soft tissues can play a role in the formation of facial asymmetry. However, given the breadth of the causes and the multiplicity of available treatment plans, it is not possible to address all these issues in this article. Hence, in this study, our focus is on chin asymmetries. A variety of developmental (such as hemifacial hypertrophy), pathological (such as tumors and cysts), traumatic (such as fractures), and functional (such as mandibular displacement) factors can cause chin asymmetry [2, 3]. So far, due to the nature and extent of asymmetry, various treatment plans have been introduced to correct these conditions [1, 4–7]. The use of prefabricated customized implants [7] and genioplasty [8] is considered the most common treatment options for the management of these abnormalities. Given the many complications reported in the use of implants such as bone resorption, infection, muscle dysfunction, hematoma, displacement, poor scarring, and dysesthesia [9–12] and the inherent technical advantages of genioplasty including stable outcomes, accurate positioning of the chin, and improvement of obstructive disorders by adjustment of muscles’ position [4, 13, 14], it seems that the use of genioplasty is a more efficient and logical treatment compared to implants.

Genioplasty is a widely used surgical procedure mainly comprising an osteotomy in the anterior lower portion of the mandible for the correction of chin deformities by allowing movement of the chin in three dimensions and positioning it in its new desired position [15]. Depending on the type of deformity, different genioplasty techniques may be used. In this article, we intend to introduce a novel computer-assisted horizontal translational osseous genioplasty technique whose main application is for the correction of chin deviation.

## Methods

**Fig. 1:**
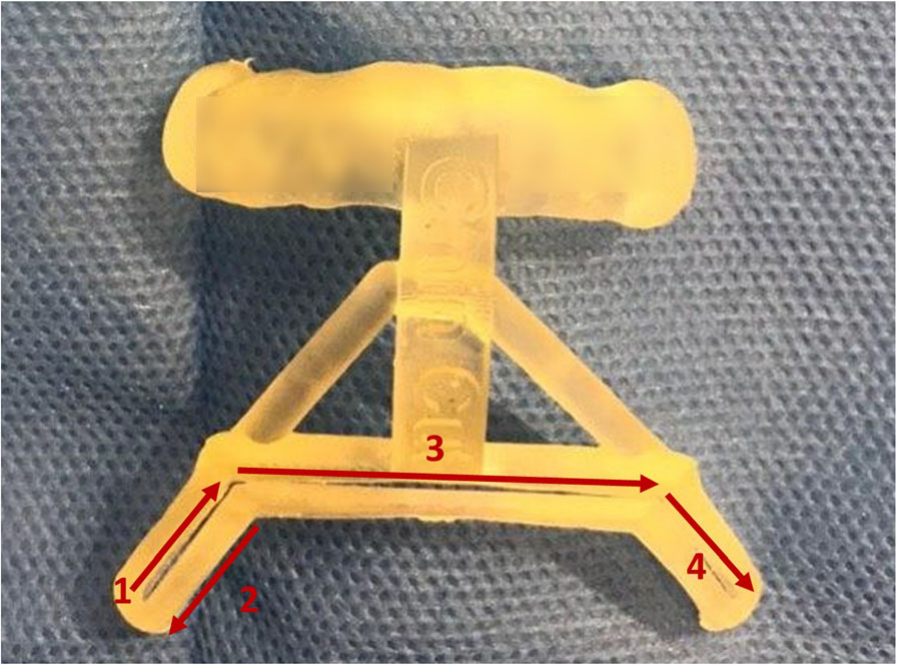
Steps of chin osteotomy using the surgical guide: (1) The first cut is made in the inclined groove at the opposite side of which the chin is deviated to. (2) The second cut is made along the outer edge of the same side. (3) In the third step, the horizontal cut is made. A bone strip from the side where the chin should be transferred to is available now. (4) By making the fourth cut, the chin is also movable, and by removing the bone strip, it can be transferred to the correct position. Finally, the same bone strip is used for the filling of the gap that was formed on the opposite side

The general concept of this technique is based on using a surgical guide to cut a bone strip from the side where the chin should be transferred to, then using the same bone strip for the filling of the bone gap that has been formed on the opposite side. In this way, by placing the dissected bone segment in the correct position and angle, the deviation of the chin and the resulting asymmetry will be eliminated. The steps of this surgical procedure are described in more detail below (Fig. 1).

**Fig. 2:**
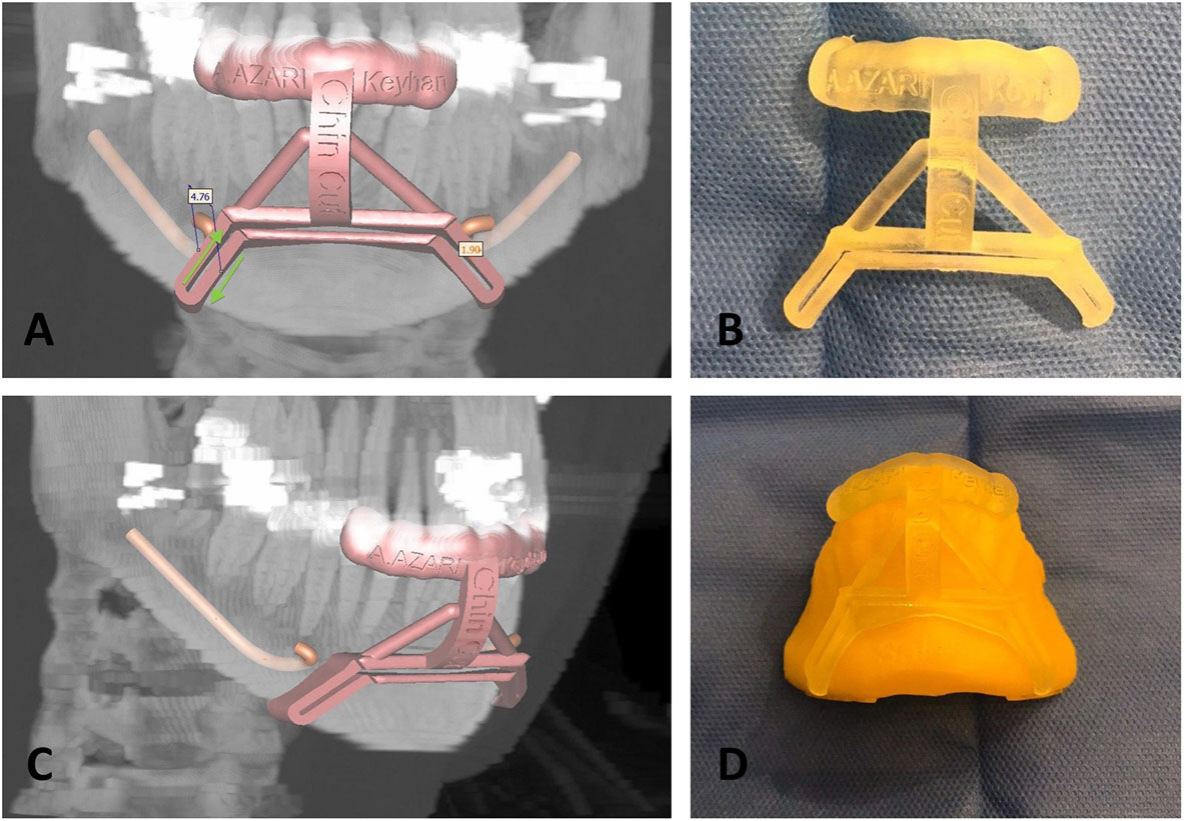
**a**, **c** Digital design of chin osteotomy surgical guide for a sample 24-year-old female patient who underwent computer-assisted horizontal translational osseous genioplasty. **b**, **d** For accurate adaptation with the chin anatomy, a clear surgical guide was fabricated using special resin through Rapid Prototyping Technology (SLA technology-RUNA CO., Tehran, Iran)

First, the three-dimensional computed tomography of the lower jaw area (1-mm incremental slices, Gantry zero, Bone Plus Protocol, HiSpeed QXi, GE Healthcare) was obtained. In order to accurately design the virtual chin osteotomy, raw data were input into KAVEH software (KAVEH Package, a special software package for Cranial and Maxillofacial Implantology/Surgery, Tehran, Iran). Eventually, a customized module was exclusively designed for each patient deformity (Fig. 2).

All patients received necessary information about the advantages, disadvantages, and potential risks of the technique and signed informed consent prior to the operation. All surgeries were performed by a single oral and maxillofacial surgeon. Patients underwent general anesthesia via nasal intubation, and following the preparation of the face and mouth, local anesthesia (2% lidocaine with epinephrine at a concentration of 1:80,000) was administered to the area. To elevate the overlying soft tissue, a vestibular incision throughout the intercanine distance at 5-mm distance from the mucogingival line was performed.

After ensuring that the surgical guide was fixed in the correct position, osteotomy was performed using a thin reciprocating saw blade. Upon completion of the osteotomy, two bone segments including a thin bone strip and a segment that comprises the main section of the chin were obtained. The path of the osteotomy lines inside the surgical guide groove and on its outer border is shown in Fig. 2a.

**Fig. 3:**
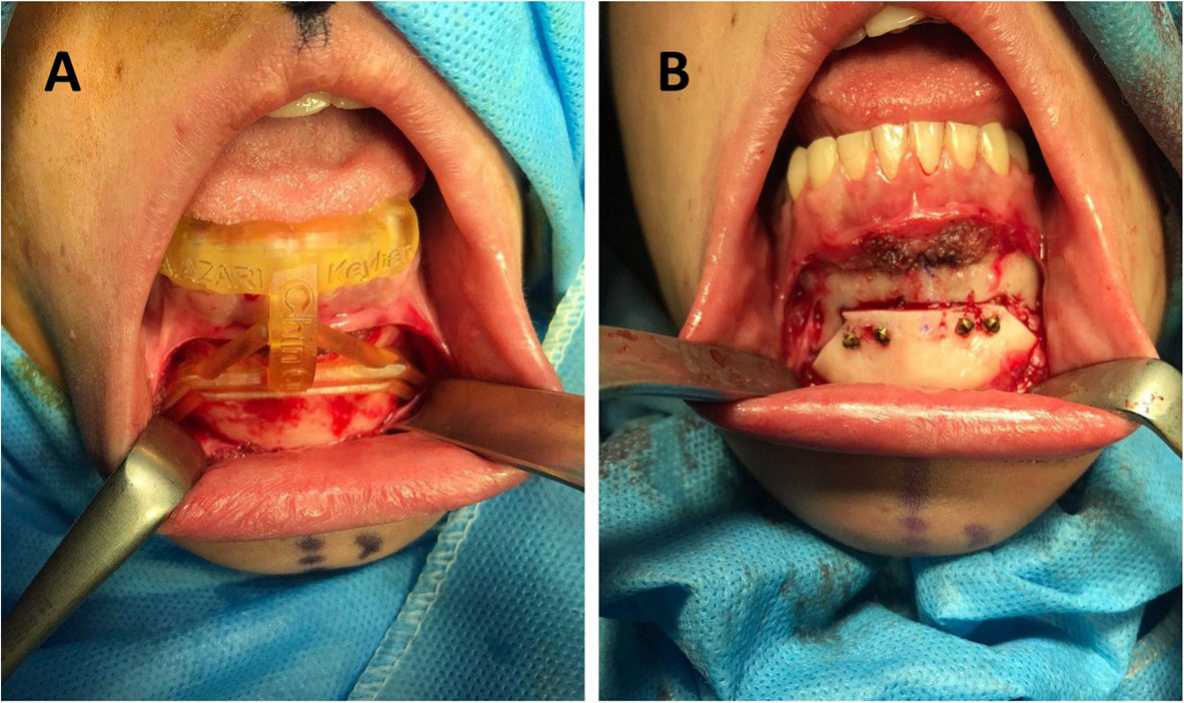
**a** Application of a chin osteotomy surgical guide in a patient with a mild deviation of the chin. **b** Fixation of the chin in the corrected position using 4 titanium screws

In the next step, the bone strip was removed from the appointed area so that the main part of the chin can be placed in the correct position following the transfer of the main segment to the correct position, and the bone strip was used to fill the gap in the opposite side. Finally, after making sure that the bone segments were in the correct position and achieving the desired symmetry, titanium screws were used to fix the bone segments in the new position (Fig. 3).

## Results

In total, 30 patients underwent the computer-assisted horizontal translational osseous genioplasty using our technique, out of whom, 24 were followed up for 1 year. Patients were assessed in terms of esthetic, symmetry, and complications in follow-up sessions.

**Fig. 4:**
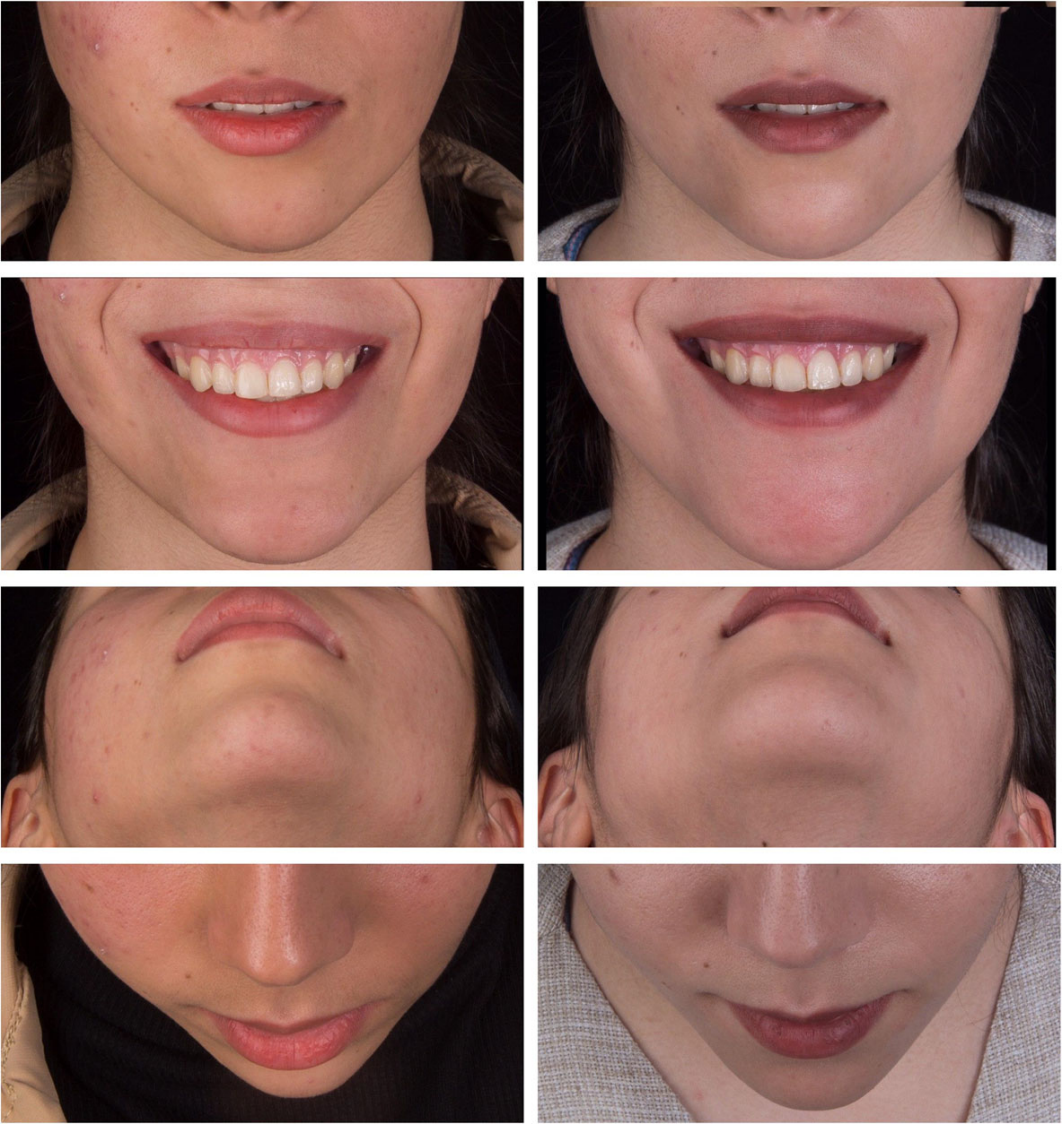
Pre-operative (left) and post-operative (right) views of a sample patient who underwent computer-assisted horizontal translational osseous genioplasty

In clinical assessments, no sign of infection, dysesthesia, muscle dysfunction, or chin deformity was observed. Compared to the maxillary anterior teeth as controls, the vitality of mandibular anterior teeth was approved using an electronic pulp tester (Parkell Inc., Farmingdale, NY, USA). Furthermore, all patients were satisfied with the final outcomes. For instance, the pre- and post-operative clinical views of a sample 24-year-old female patient who underwent computer-assisted horizontal translational osseous genioplasty are demonstrated in Fig. 4.

## Discussion

The aim of this article was to present a new computer-assisted genioplasty technique for the correction of chin asymmetries. The application of digital design technology compared to conventional techniques can provide various benefits for both surgeon and patient. Becoming more accurate and more predictable surgical outcomes and reduction in operating time are among significant advantages that are certainly of particular importance to surgeons.

Compared to previous conventional methods, this method has several advantages including more precise osteotomy and exact locating of adjacent sensitive structures such as mental nerves and roots of teeth, which is very important in reducing possible damage. However, it should be noted that the use of such a technique will not necessarily be effective in treating all deviations and asymmetries of the chin or mandible. The most important point in this regard is the comprehensive clinical and radiographic evaluation of the patient in order to accurately identify the etiology of deformity and then decision-making on the treatment plan. Certainly in cases where there are significant dental abnormalities and TMJ dysfunction, ignoring these problems and using this technique solely to correct the patient’s appearance is completely rejected.

For example, based on a patient’s problem, Singh et al. [16] proposed a treatment plan including orthodontics, sagittal split ramus osteotomy, intraoral vertical ramus osteotomy, and genioplasty to correct mandibular asymmetry in a 19-year-old patient.

Baik et al. [17] by examining a 30-year-old female patient with bialveolar protrusion, mandibular prognathism, chin retrusion, a long face, and severe facial asymmetry deducted that genioplasty alone would not be able to correct the patient’s abnormality, so they concluded that in order to improve the esthetic status, multiple complex surgical procedures should be used.

Thus, a series of procedures including anterior segmental osteotomy, LeFort I asymmetric impaction, bilateral sagittal split ramus osteotomy, 3-piece segmentation of the maxilla, anterior segmental osteotomy, advancement genioplasty, and mandibular angle contouring were performed concurrently for the patient. In contrast, the use of simpler and non-combination techniques for simpler cases is also quite common. For example, Gadre et al. [18] by using a single horizontal flip pedicled genioplasty for patients with unilateral temporomandibular joint ankylosis achieved reasonably satisfying outcomes.

In another study by Li et al. [19], the outcomes demonstrated that in patients with chin deformity, the chin template system provided a trusty method for the planning of two-piece narrowing genioplasty in a way that there were no reports of mental nerve injury, abnormal bleeding, template fracture, or difficulty in the application of the guide. In addition, all patients absolutely recovered well and were satisfied with the surgical results.

Perhaps the main limitation of this technique compared to conventional methods is the imposition of additional costs for three-dimensional computed tomography, the design of the digital treatment plan, and the fabrication of the surgical guide using Rapid Prototyping Technology.

## Conclusion

According to the authors’ experiences, it can be deduced that in patients with facial asymmetry due to chin deviation, computer-assisted horizontal translational osseous genioplasty can be used as a simple and trustworthy method to correct chin deformity. However, it seems that conducting studies with a larger sample size and longer follow-up periods will significantly reveal the strengths and flaws of this technique.

## Data Availability

The datasets generated and/or analyzed during the current study are not publicly available but are available from the corresponding author on reasonable request.

## References

[1] Chia MS, Naini FB, Gill DS (2008). The aetiology, diagnosis and management of mandibular asymmetry. Orthodontic Update.

[2] Thiesen G, Gribel BF, Freitas MPM (2015). Facial asymmetry: a current review. Dental press journal of orthodontics.

[3] Cheong Y-W, Lo L-J (2011). Facial asymmetry: etiology, evaluation, and management. Chang Gung Med J.

[4] Chang EW, Lam SM, Karen M, Donlevy JL (2001). Sliding genioplasty for correction of chin abnormalities. Archives of Facial Plastic Surgery.

[5] Keyhan SO, Cheshmi B, Fallahi HR, Asayesh MA, Fattahi T (2019). Balcony genioplasty: a novel technique for better esthetic results in patients with deep mentolabial fold. Maxillofacial plastic and reconstructive surgery.

[6] Keyhan SO, Khiabani K, Hemmat S, Varedi P (2013). Zigzag genioplasty: a new technique for 3-dimensional reduction genioplasty. British Journal of Oral and Maxillofacial Surgery.

[7] Watson J, Hatamleh M, Alwahadni A, Srinivasan D (2014). Correction of facial and mandibular asymmetry using a computer aided design/computer aided manufacturing prefabricated titanium implant. Journal of Craniofacial Surgery.

[8] Keyhan SO, Khiabani K, Raisian S, Bohlouli B, Feizbakhsh M, Hemmat S (2013). Zigzag genioplasty; patients evaluation, technique modifications and review of the literature. The American Journal of Cosmetic Surgery.

[9] Betancourt Tinoco DR, Vélez Leiva EE (2010) Mentoplastia de aumento con implante de silicona en la Clínica La Font en el periodo 1998 a 2009.

[10] Safian J (1966). Progress in nasal and chin augmentation. Plastic Reconstructive Surg.

[11] Abrahams JJ, Caceres C (1998). Mandibular erosion from silastic implants: evaluation with a dental CT software program. American journal of neuroradiology.

[12] Sciaraffia CE, Ahumada MF, Parada FJ, Gonzalez E, Prado A (2018) Bone resorption after use of silicone chin implants, long-term follow-up study with lateral chin radiography. Plastic and Reconstructive Surgery Global Open 6(7) 10.1097/GOX.0000000000001850 PMC6110682 30175015

[13] Miles BA, Leach JL (2007). Osseous genioplasty: technical considerations. Operative Techniques in Otolaryngology-Head and Neck Surgery.

[14] Park J-Y, Kim S-G, Baik S-M, Kim S-Y (2010). Comparison of genioplasty using Medpor and osteotomy. Oral Surgery, Oral Medicine, Oral Pathology, Oral Radiology, and Endodontology.

[15] Oth O, Durieux V, Orellana M-F, Glineur R (2020). Genioplasty with surgical guide using 3D-printing technology: a systematic review. J Clin Exp Dentist.

[16] Singh H, Srivastava D, Kapoor P, Sharma P (2016). Surgical orthodontic correction of mandibular laterognathism. J Orthodontic Sci.

[17] Baik U-B, Han K-H, Yoo S-J, Park J-U, Kook Y-A (2013). Combined multisegmental surgical-orthodontic treatment of bialveolar protrusion and chin retrusion with severe facial asymmetry. Am J Orthodontics Dentofacial Orthop.

[18] Gadre KS, Halli R, Shah S, Ramanojam S (2011). Horizontal flip pedicled genioplasty for correction of asymmetric chin in adult unilateral temperomandibular joint ankylosis. J Craniofacial Surg.

[19] Li B, Shen S, Yu H, Li J, Xia J, Wang X (2016). A new design of CAD/CAM surgical template system for two-piece narrowing genioplasty. Int J Oral Maxillofacial Surg.

